# Mobile phone text messaging for promoting adherence to anti-tuberculosis treatment: a systematic review

**DOI:** 10.1186/1471-2334-13-566

**Published:** 2013-12-02

**Authors:** Mweete D Nglazi, Linda-Gail Bekker, Robin Wood, Gregory D Hussey, Charles S Wiysonge

**Affiliations:** 1 The Desmond Tutu HIV Centre, Institute of Infectious Disease and Molecular Medicine and the Department of Medicine, Faculty of Health Sciences, University of Cape Town, Anzio Road, Observatory 7925, Cape Town, South Africa; 2 International Union against Tuberculosis and Lung Disease, 68 Boulevard Saint Michel, 75006 Paris, France; 3 Vaccines for Africa Initiative, Division of Medical Microbiology & Institute of Infectious Disease and Molecular Medicine, University of Cape Town, Anzio Road, Observatory 7925, Cape Town, South Africa; 4 Centre for Evidence-based Health Care, Department of Interdisciplinary Health Sciences, Faculty of Medicine and Health Sciences, Stellenbosch University, Tygerberg Campus, Francie van Zijl Drive, Tygerberg 7505, Cape Town, South Africa; 5 Division of Community Health, Department of Interdisciplinary Health Sciences, Faculty of Medicine and Health Sciences, Stellenbosch University, Tygerberg Campus, Francie van Zijl Drive, Tygerberg 7505, Cape Town, South Africa

**Keywords:** Mobile phone, Text messages, Tuberculosis treatment, Anti-tubercular agents, Adherence, Compliance

## Abstract

**Background:**

Mobile phone text messaging (SMS) has the potential to promote adherence to tuberculosis treatment. This systematic review aims to synthesize current evidence on the effectiveness of SMS interventions in improving patients’ adherence to tuberculosis treatment.

**Methods:**

We searched electronic databases (PubMed, EMBASE, Science Citation Index), reference lists of relevant articles, conference proceedings, and selected websites for eligible studies available by 15 February 2013; regardless of language or publication status. Two authors independently screened selected eligible studies, and assessed risk of bias in included studies; resolving discrepancies by discussion and consensus.

**Results:**

We identified four studies that compared the outcomes of the SMS intervention group with controls. Only one of the four studies was a randomized controlled trial. This was conducted in Argentina and the SMS intervention did not significantly improve adherence to tuberculosis treatment compared to self-administration of tuberculosis treatment (risk ratio [RR] 1.49, 95% confidence intervals [CI] 0.90 to 2.42). One of the non-randomized studies, conducted in South Africa, which compared SMS reminders to directly observed therapy short course (DOTS) reported similar rates of tuberculosis cure (62.35% vs. 66.4%) and treatment success (72.94% vs. 69.4%). A second study from South Africa, utilized SMS reminders when patients delayed in opening their pill bottles and reported increased tuberculosis cure (RR 2.32, 95% CI 1.60 to 3.36) and smear conversion (RR 1.62, 95% CI 1.09 to 2.42) rates compared to DOTS. In the third non-randomized study, conducted in Kenya, use of SMS reminders increased rates of clinic attendance on scheduled days compared to standard care (RR 1.56, 95% CI 1.06 to 2.29). Using the GRADE approach, we rate the quality of the evidence as low, mainly because of the high risk of bias and heterogeneity of effects across studies.

**Conclusions:**

This systematic review indicates that there is a paucity of high-quality data on the effectiveness of SMS interventions for improving patients’ adherence to tuberculosis treatment. The low quality of the current evidence implies that further studies (in particular randomized trials) on the subject are needed. In the interim, if the intervention is implemented outside research settings an impact evaluation is warranted.

## Background

Tuberculosis (TB) is a major public health concern, with an estimated 8.7 million incident cases and 1.4 million deaths in 2011 [1]. The burden of TB is highest in 22 low- and middle-income countries; mostly located in sub-Saharan Africa, where TB is fuelled by the HIV/AIDS epidemic [2].

The World Health Organization (WHO) guidelines for TB treatment recommend directly observed treatment short course (DOTS) strategy to monitor patient medication adherence [1,3]. This strategy includes treating TB using standardized rifampicin-based regimens of six months duration for new TB cases and eight months for retreatment cases. Failure of patients to complete TB treatment results in infectivity, drug resistance, relapse and death [4]. It is therefore important to find better ways to improving patient adherence to TB treatment.

A variety of factors may impact on patient medication adherence, and thus efforts to improve medication adherence in general are more effective when they address multiple dimensions of adherence behaviours than single-target interventions [5,6]. Several strategies for promoting TB medication adherence have been investigated. These nclude interventions promoting better health care provider-patient communication about adherence; developing or improving existing adherence support services that are offered by a multidisciplinary team (nurse, physician, pharmacy, patient etc.) [6]; directly observed therapy (which involves a health care worker, community care worker or family member directly monitoring patients swallowing their TB medication) [4]; staff motivation and supervision [4]; education and counseling [7]; reminder systems and late patient tracers to help patients keep appointments [8]; incentives and enablers [9]; contracts (that is written or verbal agreements) to return for appointment or course of treatment; and social support to assist the patient in being adherent, provided by community healthcare workers [10] or patient groups [6]. These interventions or complex combinations of the interventions may need to be employed to promote TB medication adherence.

The number of mobile phone subscriptions is growing worldwide and stood at 6.6 billion at the end of 2012 [11]. In addition, the number of unique mobile phone subscribers at the end of 2012 was estimated to be 3.2 billion, because of the use of on average 1.85 SIM cards per individual and inactive SIM connections being included by operators in their reported global mobile phone subscription totals [12]. However, the number of mobile phone users has continued to increase and is spreading to the most remote areas in the world. Evidently, the usefulness of mobile phone technology provides healthcare providers with an unprecedented opportunity to target health interventions to people who would otherwise be difficult to reach. Mobile phone text messaging, in particular the short messaging service, may enable healthcare providers to convey health information to patients and engage them in brief conversations. The short messaging service (SMS) has recently been proposed as a means of promoting TB medication adherence. For promoting adherence to TB treatment, text-messages can be sent daily or weekly to patients to remind them to take their medication [13,14] through one way communication or two-way interactive communication (i.e. patients can receive and reply to messages) [15-17]. Text-messages may also be used to notify healthcare providers that the patients have taken their medication [14,18,19]. In addition, the text message intervention can be delivered alone or bundled with economic incentives [14,19]. We therefore conducted a review of the current best evidence for the use of mobile phone text messaging to promote patients’ adherence to TB treatment [20].

## Methods

### Criteria for considering studies for this review

#### Type of studies

We planned to include only randomized controlled trials (RCTs), but later expanded the eligibility criteria to consider non-randomized studies because of the paucity of the former.

#### Types of participants

Adults (including pregnant women) or children receiving treatment for TB infection, in any setting.

#### Types of interventions

We included interventions in which mobile phone text messages were used to promote adherence to TB treatment. The text messaging had to be delivered to a patient with TB or, in the case of an infant or child, to a caregiver. We also included studies in which the intervention was compared to no intervention or other interventions for promoting adherence. We excluded studies in which used mobile phone voice speaking, voice messaging, a beeper, a pager, or multimedia messaging service as interventions. In addition, we excluded studies in which text messages are bundled with other interventions unless it was possible to separate the effects of text messaging alone.

#### Type of outcome measures

##### Primary outcomes

The primary outcome for this review was treatment adherence. We considered TB cure, successful completion of TB treatment and drug resistance development as proxies for adherence.

##### Secondary outcomes

The secondary outcomes were exposure to stigma associated with TB as a result of the SMS revealing the patient’s disease status, and patient satisfaction with the SMS intervention.

### Search methods for identification of studies

A comprehensive and exhaustive search was performed by MN with the help of an information specialist, to identify all relevant studies available by 15 February 2013 regardless of language or publication status (published, unpublished, in press, or in progress). We searched both peer-reviewed journal articles and the grey literature (non-published/internal or non-reviewed papers, reports).

### Databases

We searched the following electronic databases: PubMed; EMBASE; Cochrane Central Register of Controlled Trials (CENTRAL); ISI Web of Science (Science Citation Index); Africa-Wide Information; Cumulative Index of Nursing and Allied Health (CINAHL); and World Health Organization (WHO) library databases (WHOLIS). We used both text words and medical subject heading (MeSH) terms, in varying combinations. We adapted the search strategy to suit each database. Additional file 1 shows detailed information on the search strategy employed.

### Conference proceedings

We also searched the proceedings of the Union World Conference on Lung Health as well as the National Conference on Tuberculosis and Chest Disease (NATCON). In addition, we searched abstracts from the 2012 mHealth Summit that were published in the Journal of Mobile Technology in Medicine.

### Searching other sources

We also searched the WHO International Clinical trials Registry Platform, Clinicaltrials.gov, and the Pan African Clinical Trials Registry (PACTR) for ongoing studies. In addition, we searched the website of the mHealth Alliance and the mHealth in Low Resource Settings' resources database [21] for eligible studies. We also contacted The AIDS Support Organization (TASO) and African Medical and Research Foundation (AMREF) for information on eligible studies that we may have missed.

### Reference lists

We checked the reference lists of full-text articles assessed for inclusion in the review.

### Data collection and analysis

The methodology for data collection and analysis was based on the guidance of the Cochrane Handbook of Systematic Reviews for Interventions [22].

### Selection of studies

We developed and piloted a screening guide to ensure that the inclusion criteria are adhered to and consistently applied by all review authors. Two review authors (MN and CW), working independently, screened the titles and abstracts of all studies identified through the literature searches for eligibility. MN obtained full text of studies deemed potentially eligible by one or both authors. The two authors then independently assessed the full text of each article for eligibility, compared their results, and resolved discrepancies by discussion and consensus. For all studies excluded by the assessors, we have provided the reasons for exclusion in Table 1.

**Table 1:** Characteristics of excluded studies

**Study**	**Reason for exclusion**
**Mohammed**[27]	No appropriate control group.
**Mahmud**[33]	Text messaging intervention was delivered to community health care workers in order to improve patient-physician communication. Therefore, the study did not assess the use of SMS text messaging for promoting adherence to TB treatment in patients.
**Person**[28,29]	No appropriate control group.
**Liu**[34]	The study did not report any of the outcomes of interest.
**Kao**[30]	This is a descriptive study. No appropriate control group.
**Batra**[31]	No appropriate control group.
**Hoffman**[35,36]	A pilot study in which text messages are bundled with video messages and is not possible to separate the effects of the text messages alone.

### Data extraction and management

References were managed using Thomson ISI ResearchSoft Endnote 9.0 [23]. Two authors independently extracted descriptive and outcome data for each included article using a standardized data collection form, resolving any discrepancies by discussion and consensus. MN entered the final data into the Cochrane Collaboration Review Manager Version 5.2 statistical software [24]. CW cross-checked the data entered to ensure that there were no data entry errors.

### Assessment of risk of bias in included studies

Two authors (MN and CW) independently assessed the risk of bias in the included studies, by evaluating random sequence generation (for RCTs only), allocation concealment (for RCTs only), blinding of outcome assessors (for all studies), incomplete outcome data (for all studies), selective outcome reporting (for all studies), and other sources of bias (for all studies); in accordance with the methods by the Cochrane Collaboration [22]. Therefore, the assessment of risk of bias took into account the variation in study designs (i.e. RCTs and non-randomized studies), as certain criteria were only applicable to RCTs and others were applicable to both RCTs and non-randomized studies. Studies were scored as having a low, high, or unclear risk of bias. The two authors resolved disagreements in the assessment of risk of bias by discussion and consensus.

### Measures of treatment effect

Data analysis was conducted using the Cochrane Collaboration Review Manager Version 5.2 statistical software [24]. The outcomes of interest were all dichotomous. We calculated risk ratios (RR) and their corresponding 95% confidence intervals (CI) and p-values, when count data were available.

### Dealing with missing data

In cases of missing or incomplete information presented in included studies, we attempted to contact authors for further information.

### Data synthesis and investigation of heterogeneity

We assessed clinical heterogeneity by examining types of participants, interventions and outcomes in each study. We also assessed methodological heterogeneity by examining differences between studies in methodological factors such as the comparability of groups. All included studies were judged to be clinically and methodological heterogeneous, and we decided to preclude meta-analysis and describe findings for each study individually. Finally, we used the Grading of Recommendations Assessment, Development, and Evaluation (GRADE) approach [25,26] to assess to the quality of evidence for the effectiveness of the SMS intervention. This method results in an assessment of the quality of the body of evidence as high, moderate, low, or very low. Evidence is considered of high quality if “further research is very unlikely to change our confidence in the estimate of effect”, and moderate quality if “further research is likely to have an important impact on our confidence in the estimate of effect and may change the estimate”. Low quality evidence implies that “further research is very likely to have an important impact on our confidence in the estimate of effect and is likely to change the estimate”, and very low quality that “we have very little confidence in the effect estimate”.

### Sensitivity analyses

We did not conduct a meta-analysis, and could therefore not perform sensitivity analyses.

### Presenting and reporting of results

**Figure 1:**
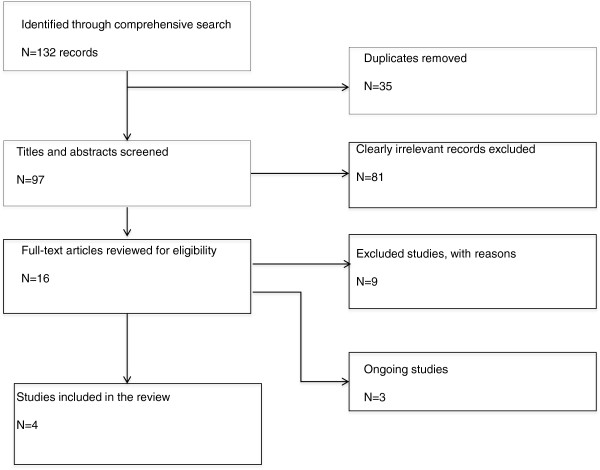
**Flow chart for studies in the systematic review.** Flow chart showing the process and results of study identification.

**Figure 2:**
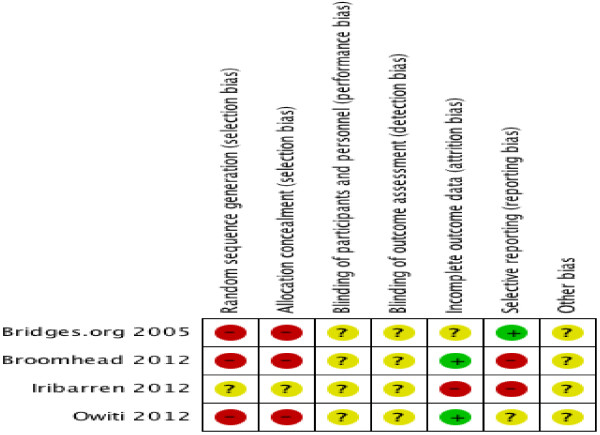
**Cochrane risk of bias summary: review authors’ judgment about each risk of bias item for each included study.** Cochrane risk of bias summary for included studies.

We have presented our findings in several ways. We have provided a flow diagram that summarizes the study selection process (Figure 1), a table of characteristics included studies (Table 2), and a risk of bias table (Table 3) and graph (Figure 2). In addition, we have provided a descriptive report of outcomes that do not have quantitative data and assessed the certainty of the evidence of effectiveness using the GRADE approach. Lastly, we have provided a list of excluded studies with reasons for exclusion (Table 1).

**Table 2:** Characteristics of included studies

**Study ID**	**Bridges.org **[37]
**Methods**	The evaluation used both qualitative and quantitative data collection methods. Structured interviews using a questionnaire were conducted among patients and staff. Additional information was collected from patient records, background documents and reports and clinic visits.
**Participants**	Patients, clinic staff, TB experts and managers at the City of Cape Town Health Directorate.
**Interventions**	Daily SMS reminders were used to remind patients who self-administer their medication (i.e. not on DOTS) to take their drugs.
**Outcomes**	1) Health Outcomes serve as a proxy for TB treatment adherence
	On Cue Compliance service (from 221 patients with records available):
	Cure rate = 62.35%
	Completion rate = 10.59
	Treatment success rate = 72.94%
	Clinic-based DOTS (in particular new smear-positive TB patients in the third quarter of 2003):
	Cure rate = 66.4%
	Completion rate = 3.0%
	Treatment success rate = 69.4%
	Health outcomes between groups were similar.
	2) Patient satisfaction with the SMS intervention
**Notes**	
**Study ID**	**Broomhead**[38]
**Methods**	A retrospective analysis comparing the costs and health outcomes of the DOTS-SIMPill cohort with DOTs-only controls.
**Participants**	24 New smear-positive TB patients who presented to Betty Gaetsewe Clinic and commenced the 6-month treatment on first line and anti-TB medication enrolled for SMS based medical adherence support (MAS) pilot in 2005 and 96 DOTs-only control patients presenting for the months during the pilot was running.
	Frequency matching was used to match MAS pilot participants with controls in a 4:1 ratio. Matching was on TB treatment, local clinic, gender and age.
**Interventions**	Intervention:
	MAS system. It consists of a device that attaches to the standard pill bottle or blister pack and sends an SMS every time the patient opens the bottle to a Web-base application. This is taken as a proxy for TB treatment adherence.
	Control:
	DOTS-only controls
**Outcomes**	Health outcomes (i.e. smear conversion rate and TB cure rate) served as a proxy for TB treatment adherence
	MAS group:
	Smear conversion rate = 62.5%
	TB cure rate = 75.0%
	Control group:
	Smear conversion rate = 38.4%
	TB cure rate = 32.3%
	Both the smear conversion rate and TB cure rate were significantly higher for the MAS group compared with the control group
	Smear conversion rate: RR 1.62 (95% CI 1.09-2.42)
	TB cure rate: RR 2.32 (95% CI 1.60 – 3.36)
**Notes**	
**Study ID**	**Iribarren**[40]
**Methods**	A parallel design randomized control pilot study
**Participants**	37 newly diagnosed TB patients (18 in the intervention group and 19 in the control group)
**Interventions**	Intervention:
	Standard Care plus a SMS-based intervention which included instructing patients to “text in” after self-administration of medication; reminders/check-in when patient did not “text in”; receipt of bi-weekly SMS education messages; and the option to consult during the first two months intensive treatment phase.
	FrontlineSMS network was employed.
	Control:
	Self-administration of TB treatment (standard of care)
**Outcomes**	Of the intervention group, 77% (22%-100%) notified (i.e. self report via text message) that they took their medication over a 60 day period. The control group was asked to complete medication calendars over the same period but only 53% of them returned the calendars. We found that the SMS intervention did not statistically improve adherence to TB treatment (RR 1.49 [95% CI 0.90-2.42]).
**Notes**	Additional information obtained from the primary author. The full article for the corresponding conference abstract is yet to be published.
**Study ID**	**Owiti**[39]
**Methods**	A feasibility pilot study
**Participants**	187 TB patients with mobile phones
**Interventions**	Intervention:
	Receiving text messages in Ki-Swahili which were delivered one day prior to the patients’ clinic appointment
	Control:
	Not receiving text messages (due to technical reasons)
	Clinic attendance on scheduled days
**Outcomes**	• Received at least one text: 101/150
	• Did not receive a text (due to technical reasons): 16/37
	RR 1.56 [95% CI 1.06-2.29]; p-value <0.0007.
**Notes**	• We noted an error in the table presented by the authors which occurred in the rows for males and females, in particular, the cell containing data for males who did not receive a text message were transposed with that containing data for females who received at least one text message. However, we did not use that information. Instead, we used the data in the total row, that was corroborated with the information in the abstract text.
	• The full article for the corresponding conference abstract is yet to be published (Dr P. Owti, personal communication)

**Table 3:** Risk of bias in included studies

**Study ID**	**Bridges.org **[37]	
**Bias**	**Authors judgment**	**Support for judgment**
**Blinding of participants and personnel (performance bias)**	Unclear risk	Blinding of participants and study personal were not reported.
**Blinding of outcome assessment (detection bias)**	Unclear risk	The blinding of outcome assessors was not specified.
**Incomplete outcome data (attrition bias)**	Unclear risk	88/309 missing from the intervention group; missing data are not reported for the control group. It remains unclear whether the proportion of missing data was balanced across groups
**Selective reporting (reporting bias)**	Low risk	The outcome reporting in the study report was comparable with the outcomes pre-specified in the methods.
**Other bias**	Unclear risk	Given the observational nature of the study there might be confounding variables that were not accounted for in the analysis (comparisons for health outcomes which serve as a proxy for TB treatment adherence)
**Study ID**	**Broomhead [**[38]	
**Bias**	Authors judgment	Support for judgment
**Blinding of participants and personnel (performance bias)**	Unclear risk	Blinding of participants and study personal were not reported.
**Blinding of outcome assessment (detection bias)**	Unclear risk	The blinding of outcome assessors was not specified.
**Incomplete outcome data (attrition bias)**	Low risk	No missing data in both the intervention and the control group.
**Selective reporting (reporting bias)**	High risk	Comparisons for health outcomes (i.e. smear conversion rate, cure rate and MDR TB rate) mentioned in text in the results but only smear conversion rate and cure rate (with significant results) were reported in the table.
**Other bias**	Unclear risk	Given the observational nature of the study there might be confounding variables that were not accounted for in the analysis (comparisons for health outcomes which serve as a proxy for TB treatment adherence)
**Study ID**	**Iribarren**[40]	
**Bias**	**Authors judgment**	**Support for judgment**
**Random sequence generation**	Unclear risk	The random sequence generation process was not described.
**Allocation concealment**	Unclear risk	The method of concealment was not described.
**Blinding of participants and personnel (performance bias)**	Unclear risk	Blinding of participants and study personal were not reported.
**Blinding of outcome assessment (detection bias)**	Unclear risk	The blinding of outcome assessors was not specified
**Incomplete outcome data (attrition bias)**	High risk	Additional information obtained from the primary author revealed that no data are missing for the intervention group and 9/19 for the control group. Reasons for missing data were due to non-responsiveness of the intervention group.
**Selective reporting (reporting bias)**	High risk	Initial efficacy outcomes (notification rates and sputum conversion rates) and patient acceptability were mentioned in the methods, but only patient notification rates, follow sputum smear culture and patient acceptability reported in the results. Additional obtained from the primary author revealed that data on the final outcomes are yet to be collected and published.
**Other bias**	Unclear risk	The lack of description of the random sequence generation process and the method of concealment suggests that there might be confounding variables that were not accounted for in the analysis (comparisons for TB treatment adherence)
**Study ID**	**Owiti**[39]	
**Bias**	**Authors judgment**	**Support for judgment**
**Blinding of participants and personnel (performance bias)**	Unclear risk	Blinding of participants and study personal were not reported.
**Blinding of outcome assessment (detection bias)**	Unclear risk	The blinding of outcome assessors was not specified
**Incomplete outcome data (attrition bias)**	Low risk	No missing data in those receiving text reminders and not receiving text reminders
**Selective reporting (reporting bias)**	Unclear risk	Inadequately information provided as this was a conference abstract.
**Other bias**	Unclear risk	Given the observational nature of the study there might be confounding variables that were not accounted for in the analysis (comparisons for scheduled clinic appointment attendance which serves as a TB treatment adherence)

## Results

### Study flow and description of studies

The process and results of study identification are outlined in a flow diagram (Figure 1). We identified 132 records through a comprehensive search. We removed 35 duplicates and screened 97 titles and abstracts. We excluded 81 clearly irrelevant records, and reviewed 16 full-text articles and abstracts for eligibility. Among the potentially eligible records, seven studies reported in nine publications were excluded with reasons given below, four studies met our inclusion criteria, and three are ongoing studies.

### Excluded studies

We obtained the full text of 16 potentially eligible articles; from which we excluded 9 articles, representing 7 individual studies. Studies conducted by Mohammed and colleagues [27], Person and colleagues [28,29], Kao and colleagues [30] and Batra and colleagues [31] were excluded because of the absence of an appropriate control. The study by Mahmud and colleagues [32,33] was excluded because the text messaging intervention was delivered to community healthcare workers rather than patients. The study by Liu and colleagues [34] was excluded because it did not mention any outcomes considered in this review. The study by Hoffman and colleagues [35,36] was excluded because text messages were bundled with video messages and it was not possible to separate the effects of text messaging alone. We provide the reasons for excluding each of these publications in Table 1.

### Included studies

We provide detailed information of included studies in Table 2, and summarize key features below.

The non-governmental organization Bridges.org conducted a pilot study in South Africa in which daily SMS reminders were used to remind patients who self-administer their medication (i.e. not on DOTS) to take their drugs [37]. The pilot study started in January 2002 and as of March 2005 had enrolled over 300 new smear positive TB patients at the clinic, with more than 280 having completed their 6-month course of anti-tuberculosis therapy (or 8-month course for retreatment TB). Health outcome data were only available for 221 TB patients. The health outcomes data for the 221 TB patients receiving SMS reminders were compared with that of controls (all smear-positive patients attending clinic-based DOTS who had health outcomes data for the third quarter of 2003). Bridges.org did not report the number of controls. This precluded the calculation of a risk ratio and its confidence intervals for this review. Bridges.org found the SMS intervention and the clinic-based DOTS groups were similar with regard to rates of TB cure (62.35% vs. 66.4%) and treatment success (72.94% vs. 69.4%); but the rate of completion of TB treatment was slightly higher in the SMS intervention group compared to the clinic-based DOTS group (10.59% vs. 3.0%). Bridges.org believed that the pilot produced similar results that are normal for the clinic but did not demonstrate a significant improvement. Numerous issues were attributed to the lack of additional benefit of the SMS reminders. These included problems in monitoring treatment adherence; lack of ownership of the service at the clinic due to the lack of proactive participation of staff; lack of regular feedback and interaction among all stakeholders and the fact that a significant number of patients failed to use the service as instructed.

Bridges.org also evaluated patient satisfaction with the SMS intervention among 26 participants using structured questionnaires. The study authors found that most patients were satisfied with the SMS reminders. The patients reported feeling “good to think nurses at the clinic care enough to SMS you every morning” and feeling “more connected to the clinic than usual”, and one patient felt that she/he “would definitely forget to take my tablets if I didn't get an SMS from the clinic [37].

Broomhead and Mars [38] conducted a retrospective analysis of a 2005 pilot study of smear positive TB patients commencing a 6-month course of anti-tuberculosis therapy in South Africa, in which health outcomes in patients given a wireless pill bottle (SIMpill®) that sends an SMS to a central server notifying it of the patient taking their medication (plus standard DOTS) were compared with matched controls who received standard DOTS only. Frequency matching was utilized to match participants from the pilot study with controls in a 1: 4 ratio. We analyzed participants according to whether or not they received SMS reminders. Rates of sputum smear conversion and TB cure were provided for two scenarios. Scenario one included all 24 participants who took part in the pilot and their 96 controls. Scenario two excluded 6 participants that took part in the pilot but who subsequently died, along with their 24 matched controls, thereby reducing the cohorts to 18 participants from the pilot and their 72 controls. We used health outcomes data from scenario one for this systematic review. The study found significantly higher sputum smear conversion rates among those who received standard DOTS in combination with SIMpill® than those who received only standard DOTS (RR 1.62, 95% CI 1.09 to 2.42). The study also found significantly higher TB cure rates in the SIMpill® - DOTS group than in the DOTs only group (RR 2.32, 95% CI 1.60 to 3.36). These findings suggest that there was improved treatment adherence when using SIMpill® in combination with standard DOTS than when using standard DOTS alone [38].

Owiti and colleagues [39], in a pilot feasibility study from Kenya, assessed the use of SMS reminders to improve clinic appointment compliance. Rates of scheduled clinic appointment attendance were compared between patients receiving and those not receiving (for technical reasons) SMS reminders. The text messages were sent in Ki-Swahili one day prior to their clinic appointments using Frontline SMS platform. We took the rate of clinic appointment attendance on scheduled days as a proxy for adherence. In this study, 150 patients received at least one SMS reminder and 37 did not receive an SMS. Those who received an SMS reminder were 1.6 times more likely to adhere to scheduled clinic appointment compared to those who did not (95% CI 1.06 to 2.29) [39].

Iribarren and colleagues [40], in a pilot parallel design randomized control study among newly diagnosed TB patients commencing anti-tuberculosis treatment in Argentina, randomly assigned 19 patients to standard care (self-administration of medication) and 18 to the intervention arm. The intervention arm received standard care and a SMS-based intervention which included instructing patients to “text-in” after self-administration of medication; delivery of SMS reminders when patient did not “text in”; and receipt of a bi-weekly SMS providing educational information and the option to consult during the first two-month intensive treatment phase. Educational text messages were selected based on the Informational-Motivational-Behavioral Skills Model. Text messages were sent using FrontlineSMS, which is a free automated SMS platform. The outcome of interest for this systematic review was self-reported adherence (as measured using notification rates over a 60-day period for the intervention arm and receipt of medication calendars at 60 days for the control arm). The analysis was by intention to treat. At 60 days, patients in the intervention group had a higher self-reported adherence rate than those in the control group, but this difference was not statistically significant (RR 1.49, 95% CI 0.90 to 2.42) [40].

### Risk of bias in included studies

We considered the methodological quality of the included studies to be generally poor. Three studies were observational in nature and one was a pilot randomized controlled trial. In two studies we used treatment outcomes as a proxy for treatment adherence while in the remaining two, we assessed treatment adherence by notification rate and clinic appointments respectively. For random sequence generation, the RCT was judged to have an unclear risk of bias because the study did not describe the process used to generate the randomization sequence. For allocation concealment, the RCT was judged to have an unclear risk of bias because the method used to conceal treatment allocation was not described. The blinding of participants and personnel was not reported in the RCT and the study was judged to have an unclear risk of bias for this component. The blinding of outcome assessors was not specified in any of the studies and the risk of bias being introduced to the studies at the time of outcome assessment was judged to be unclear. Regarding completeness of outcome data, one study was judged to have an unclear risk of bias because it was unclear whether the proportion of missing data was balanced across intervention groups. Two studies were judged to have a low risk of bias because there were no missing data. One study was judged to have a high risk of bias because no data were missing in the intervention group but 47.3% (9/19) of data were missing in the control group. This information was obtained from email communication with the study authors. Two studies had selective reporting of outcomes and were judged to have a high risk of bias. One study had inadequate information provided on selective outcome reporting and was judged to have an unclear risk of bias, while another had no selective outcome reporting and was judged to be at low risk of bias. Given the observational nature of three of the included studies, there might be other confounding variables not accounted for in the analyses. Therefore, these studies were not judged to be free from other sources of bias. For the included RCT, the lack of description of the random sequence generation process and the method of allocation concealment suggests that there might be other confounding variables not accounted for in the analysis. We provided a detailed analysis of the risk of bias in Table 3 and Figure 2.

### Effects of intervention

A meta-analysis of data from included studies was not done because of significant clinical and methodological heterogeneity among the studies. Findings for each study are described individually. Bridges.org 2005 [37] found that the SMS intervention and the clinic-based DOTS groups were similar with regard to rates of TB cure (62.35% vs. 66.40%) and treatment success ( 72.94% vs. 69.4%). However, the rate of completion of TB treatment was higher in the SMS intervention compared to the clinic-based DOTS group (10.59% vs. 3.00%). Broomhead [38] showed a higher TB cure rate among patients receiving standard DOTS plus SIMpill® than those receiving standard DOTS only (RR 2.32, 95% CI 1.60 to 3.36). The same study also found a higher sputum smear conversion rate among patients receiving standard DOTS plus SIMpill® compared to those receiving standard DOTS only (RR 1.62, 95% CI 1.09 to 2.42). Owiti [39] found increased rates of clinic attendance on scheduled days among patients receiving SMS reminders compared to those who did not (RR 1.56, 95% CI 1.06 to 2.29). Iribarren [40] found that self-reported adherence was higher the SMS-based intervention group than in the standard care group, but this difference was not statistically significant (RR 1.49, 95% CI 0.90 to 2.42).

### Ongoing studies

As indicated earlier, we found three ongoing RCTs. An RCT to evaluate the impact of SMS text messages on adherence to treatment for latent TB is currently ongoing in British Columbia, Canada [41]. In Karachi, Pakistan, an RCT is currently underway to assess the impact of interactive reminders on drug compliance and treatment outcomes [42]. Another RCT is currently underway in China to assess the impact of mobile phone text messages on TB treatment adherence [43]. We provide more information on these ongoing RCTs in Table 4.

**Table 4:** Characteristics of ongoing studies

**Item/study**	**Lester **[41]	**Mohammed **[42]	**Jiang **[43]
**Trial name or title**	A Randomized Controlled Trial to Examine the Effectiveness of Use of Mobile Phones and Text Messaging to Improve Adherence to Treatment of Latent TB	Monitoring Patient Compliance with Tuberculosis Treatment Regimens	Cluster randomized trial of using mobile text messaging and a medication monitor in tuberculosis (TB) case management
**Methods**	Open-label multicenter randomized controlled trial	Allocation: Randomized, Intervention Model: Parallel Assignment, Masking: Open Label, Primary Purpose: Supportive Care.	Cluster Randomized Controlled Trial
**Participants**	Subjects initiating treatment for latent TB infection who are aged above 18 years, who own a mobile phone or share mobile phone access with a household member who consents to participate. In addition, the subject should be able to read text-messages in English or has a family member or friend that can provide translation and assistance with text-messages during the duration of the study	Inclusion criteria:	Inclusion criteria:
		• New, spear-positive drug susceptible TB patients who have been on treatment for less than two weeks	• TB patients, smear-positive or smear-negative, recruited from the study clusters (county/district)
		• 15 years and older	• Willing to participate in the study
			• Conscious without any mental disease
		• Access to a mobile phone (self-report)	
			**•** Conscious without any visual, auditory or language impairment
			**•** At least 18 years old
		• Intending to reside in Karachi for the duration of treatment	**•** Patient or family member is able to read a short message service (SMS)/ text and use medication monitor after training
		Exclusion criteria:	
			Exclusion criteria:
			**•** Does not meet inclusion criteria
		• Patients who do not have regular access to a mobile phone	**•** Patients with tuberculosis pleurisy
			**•** Patients with no sputum smear data at tuberculosis diagnosis
		• Patients who have previously received treatment	
		Patients who have another member in their household who is already a part of the study	
**Interventions**	Weekly text messages will be sent to the participants in the intervention arm asking them how they are	Other: Interactive Reminders	This is a cluster randomized non blinded trial. Clusters are defined as a county or district. This is a four armed trial, three intervention arms and one control arm:
		Daily SMS reminders sent to TB patients at a pre-specified time. They are asked to respond to the reminders. If a response is not received within two hours, they are sent another reminder for up to three hours per day	
			1. Mobile phone
			Patients are provided with mobile phones as a reminding tool to take their tuberculosis medication. On medication intake days patients are sent a SMS to remind them to take their medication. They respond with a brief message when medication is taken. Doctors in TB dispensary collect the SMS feedback from patients to assess how many doses are missed in a month. Based on the missed doses, additional intervention and incentive mechanisms are implemented such as visits from the township/village doctor and incentives per visit given to the township/village doctor.
			2. Medication monitor
			Patients are provided with a medication monitor box with reminding functions. This tool is used to remind patients to their tuberculosis medication and also records drug intake. Doctors at the TB dispensary collect the drug intake record from medication monitor monthly to assess that how many doses are missed in a month. Based on the missed doses, additional intervention and incentive mechanism are implemented as described in the mobile phone intervention (1)
			3. Mobile phone and medication monitor
			Patients are provided with both the mobile phone and medication monitor box with reminding function for as tools for communication, reminding and recording drug intake. The drugs intake record from medication monitor and SMS from patients are collected monthly, and the number of doses missed in a month is calculated using the drug intake record of the medication monitor. Based on the missed doses, additional intervention and incentive mechanism are implemented as described in the mobile phone intervention (1)
			4. Control
			Patients are managed based on the current standard of care.
			All patients will be followed up to the end of tuberculosis treatment.
**Outcomes**	Primary outcome: Successful completion of LTBI treatment regimens. [Time Frame: 4 or 9 months]. Successful treatment completion is defined as taking at least 80% of the doses of INH prescribed within 12 months or at least 80% of the disease of RIF prescribed within 6 months	Primary outcomes:	Primary outcome:
		• Treatment Outcomes [Time Frame: After 6 to 8 months of treatment] [Designated as safety issue: No]. The investigators will compare clinically reported treatment outcomes between the intervention and control groups.	
			• The mean proportion of months on TB treatment where at least 3 doses were missed in a month (this is based on pill count data from the medication monitoring box)
		• Sputum conversion [ Time Frame: At 2, 5, and 6/7 months of treatment ] [ Designated as safety issue: No ]The investigators will look at sputum test results for patients at months 2, 5, and 6/7 of their treatment to compare when sputum conversion occurs between the intervention and control group at these three periods during their treatment.	
			Secondary outcomes:
			• The mean proportion of months a patient has at least 7 doses missed
			• The mean proportion of overall missed doses
		• Treatment compliance [Time Frame: Monthly visits for 6 to 8 months of treatment ] [ Designated as safety issue: No ]The regularity of treatment will be measured using urinalysis tests that detect the presence of isoniazid or rifampicin, a first line drug for TB treatment, in patients' urine. These results will be collected through monthly "surprise" visits to the participants' houses. The number of negative results will be compared between treatment and control groups.	
			• Proportion of patients defined as non-adherent (at least 10% of doses missed)
			• Proportion of patients defaulting during TB treatment
			• Proportion of smear positive TB cases who become smear negative at 2 months
		Secondary outcomes:	
		• Physical fitness and mobility [Time Frame: Monthly visits for 6 to 8 months of treatment] [Designated as safety issue: No].The investigators will measure physical fitness and mobility through questionnaires conducted with patients during household visits each month that they are on treatment. The investigators are using two indices. The physical fitness index will record respondents’ ability to perform certain tasks. The mobility index will record the mobility of participants.	
			• The proportion of patients with treatment outcome of cure or completed treatment
		• Psychological Impacts [ Time Frame: Monthly visits for 6 to 8 months of treatment ] [ Designated as safety issue: No ]In order to gauge the psychological impacts of the system, the investigators will be looking at participants' perceptions on the likelihood of being cured, how they feel on a given day using the pain scale, and how supported they feel. This data will be collected through questionnaires conducted at each monthly mid-line visit.	
**Starting date**	April 2012	2011	2011
**Estimated study completion date**	December 2014	November 2014	August 2012
**Contact information**	Richard Lester: richard.lester@bccdc.ca Natasha Van Borek: natasha.vanborek@bccdc.ca	Shama Mohammed: shama.mohammed@irdresearch.org	Professor Shiwen Jiang: jiangsw@chinatb.org
**Notes**	ClinicalTrials.gov identifier: NCT01549457	ClinicalTrials.gov identifier: NCT01690754	Current Controlled Trials identifier: ISRCTN46846388

## Discussion

Four studies with a total of 565 participants were included in this systematic review. Three studies were observational in nature and only one was a randomized controlled trial. A meta-analysis could not be performed because there was significant clinical and methodological heterogeneity in the included studies. Overall, the included studies suggest that patients receiving mobile phone text messaging interventions had rates of adherence to TB treatment comparable to or higher than those receiving no intervention. Therefore, the findings provide mixed evidence for the effectiveness of mobile phone text messaging interventions designed to promote adherence to TB treatment.

Though the evidence is mixed, we cannot ignore the potential of mobile phone text messaging to transform the delivery of health messages to patients. Mobile phones have spread globally; 45% of the world’s population were estimated to have access to a mobile phone at the end of 2012 [12,44]. In addition, the use of SMS has become popular throughout the world. Globally, there were an estimated 5.9 trillion SMS messages sent in 2011 and SMS traffic is expected to reach 9.4 trillion messages by 2016 [45,46]. This increasingly popular mode of communication can be used to deliver short health messages to people anywhere and provide interactive feedback and support to people when they need it the most. Previous research has shown that SMS interventions are effective as a means to promote multiple healthy behaviors such as adherence to antiretroviral treatment [13], diabetes management and control [47], smoking cessation [48], and immunization compliance [49-51]. We have found that currently available research utilizing SMS interventions to promote adherence to TB treatment is inconclusive.

### Overall completeness and applicability of evidence

The strength of this systematic review lies in our adherence to international standardized guidelines on the conduct and reporting of systematic reviews [22]. However, due to paucity of published data, the majority of studies in our sample were from the grey literature. Only one of the four included studies was peer-reviewed. One of the remaining three was a non-published report and two were conference abstracts. These publications have not undergone peer review and may therefore include selective reporting biases. Only one study included in this review was an RCT and the others were observational in nature.

### Quality of the evidence

Knowledge of the best available evidence is essential for policymakers, patients and clinics in making informed health care decisions. We used the GRADE system [25,26] to assess the quality of evidence in this review. Overall, the quality of the available evidence on the use of SMS interventions to promote patient TB treatment adherence is low. The implication of the low-quality evidence is that further improved quality research (in particular RCTs) is needed to enhance our level of certainty in the effectiveness of SMS interventions to improve adherence to TB treatment. The main reasons for rating down the quality of evidence were a high risk of bias and heterogeneity of effects across included studies.

### Potential biases in the review process

Although this systematic review adheres to the standardized guidelines of conduct and reporting of systematic reviews [22], there are certain limitations. Although we did not set out to exclude non-English studies in this review, non-English studies may have been missed because they are less commonly indexed in the selected databases compared to English studies. In addition, unpublished eligible studies may have been missed. Factors which tend to influence publication are statistically significant results, size of the study, funding, prestige, type of design and study quality [52]. We found that the majority of publications were retrieved from the grey literature, and these publications may have been systematically different from those in journal articles, which have undergone scrutiny prior to publication. We have also, due to the paucity of available research, included studies that were pilot or feasibility studies. These studies often have numerous design issues, including small sample sizes, poor reporting, and lack of an appropriate control group [53]; which may influence the size of the effect estimate and the quality of the evidence.

### Agreement or disagreements with other studies or reviews

A previous narrative review by Denkinger et al. [54] has summarized the recent developments at the intersection of TB care and control and m-Health, including evidence from PubMed, grey literature mentioned in the reference lists of articles, and additional studies that were identified from experts in the field. Our systematic review has a particular focus on the use of SMS interventions to promote patient adherence to TB treatment, is more up to date, and provides evidence-based conclusions.

### Comparison to the literature on the use of mobile phone text messaging for improving adherence to antiretroviral medication

There is very little peer-reviewed literature available on the use of mobile phone text messaging to improve anti-tuberculosis medication adherence. In contrast, there is relatively more peer-reviewed literature available on the use of SMS-based platforms for improving adherence to antiretroviral treatment [13,55] A systematic review published in 2012 that included two RCTs on the use of mobile phone text messages to promote antiretroviral medication adherence found that weekly mobile phone text messaging significantly improved adherence to antiretroviral medication in HIV infected patients, compared to standard care [13]. However, an RCT from Cameroon published in the same year that was not included in the aforementioned systematic review found that standardized motivational text messaging did not significantly improve adherence to antiretroviral treatment in HIV-infected patients, compared to standard care [55]. Studies investigating the use of SMS interventions as an aid to promoting adherence to medication in resource-limited settings will need to take into account barriers such as low-literacy levels; language barriers; lack of access to the owner of a mobile phone given that sharing of mobile phones is common in many places; restrictions on the content of text messages; issues such as habituation and the ignoring of messages when text messages are delivered too frequently; privacy and disclosure issues; poor mobile phone service provision [17,56,57]; the inability of mobile phone users to charge their phones due to lack of electricity; the inability to buy pre-paid phone cards; and the high incidence of mobile phone theft and phone number changes in some parts of the world.

### Implication for practice

Whilst some of the formative research presented here as part of this systematic review of mobile phone text messaging and its efficacy in enhancing adherence in TB programs shows promise, and although this intervention is likely to be light on resources, the inconclusive evidence of efficacy implies that this intervention should not yet be scaled up in TB programs outside research settings.

### Implication for research

The paucity of data and the imprecise methodology encountered in the research in this field to date suggest that there is room for more carefully designed research. Future studies should have standardized outcome measures such as TB cure, successful completion of TB treatment, and development of drug resistance. Considered in this systematic review but not found, is the potential for stigmatization and disclosure that are inherent risks in this kind of intervention. These (perceived) risks may undermine efficacy and acceptability and should therefore be studied. In addition, future studies need to pay attention to the reporting of the delivery of the intervention i.e. length of messages, content of messages, timing of messages, frequency of messages, SMS platform used to send messages (automated versus manual), and two-way versus one-way communication. These factors may influence efficacy, acceptability, cost, and scale up. Although mobile phone text messaging is attractive in that it is relatively inexpensive, cost of development, implementation and maintenance needs to be considered and weighed against effectiveness. In addition, since RCTs are the gold standard for testing the effect of an intervention, it would be useful to see more RCTs on this subject. It is encouraging that three RCTs are currently underway in Canada, Pakistan and China [41-43].

## Conclusions

The findings of this systematic review indicate that SMS interventions have a potential for use to improve patients’ adherence to TB treatment, though the evidence is inconclusive. To conclude that such an intervention is effective is difficult because there is a paucity of high-quality studies. The current evidence is of low quality implying that further research is very likely to have an important impact on our confidence in the effectiveness of this intervention and is likely to change the magnitude of the estimate of effect. The results of the systematic review also lay an important foundation on which future studies can build upon. Future studies (in particular RCTs) should be of robust methodology, well reported and interventions optimized in order to establish their benefit. We will update this systematic review when new evidence emerges from the three ongoing studies.

## Abbreviations

CENTRAL: Cochrane Central Register of Controlled Trials; CINAHL: Cumulative index of nursing and allied health; DOTS: Directly observed treatment short course; GRADE: Grading of recommendations assessment, development, and evaluation; MeSH: Medical subject heading; NATCON: National Conference on Tuberculosis and Chest Disease; PACTR: Pan African Clinical Trials Registry; RCT: Randomized controlled trial; SMS: Short messaging service; TB: Tuberculosis; WHO: World Health Organization; WHOLIS: World Health Organization (WHO) library databases; TASO: The AIDS Support Organization; AMREF: African Medical and Research Foundation.

## Competing interests

The authors declare that that they have no competing interests.

## Authors’ contributions

MN and CW contributed to the conception and design of the review, and were involved in data acquisition. MN analyzed the data with input from all co-authors, and all authors participated in the interpretation of the results. All authors were involved in the drafting of this systematic review article and have given their approval for publication.

## Pre-publication history

The pre-publication history for this paper can be accessed here:

http://www.biomedcentral.com/1471-2334/13/566/prepub

## Supplementary Material

Additional file 1 Search strategies for databases. Click here for file
