# Supplementary material for: Mobile phone text messaging for promoting adherence to anti-tuberculosis treatment: a systematic review
Source: BMC Infect Dis. 2013 Dec 2;13:566. doi: 10.1186/1471-2334-13-566 (PMC4219402; doi:10.1186/1471-2334-13-566)
Supplement: Additional file 1 — Search strategies for databases. [file 1471-2334-13-566-S1.doc]

**Table 1. Search methods for PubMed conducted on 15 February 2013**

| **Number** | **Search Terms** | **Hits** |
| --- | --- | --- |
| #4 | #1 AND #2 AND #3 | 55 |
| #3 | “medication adherence” [MeSH] OR “patient compliance”[MeSH] OR adherence [tiab] OR compliance [tiab] | 154484 |
| #2 | "cellular phone"[MeSH] OR "reminder systems"[MeSH] OR telemedicine[MeSH] OR "wireless technology"[MeSH] OR "text messaging"[MeSH] OR text*[MeSH] OR "medical informatics applications"[MeSH] OR SMS[tiab] OR MMS[tiab] OR "mobile phone"[tiab] OR mHealth[tiab] OR "mobile health"[tiab] | 313184 |
| **#1** | **"tuberculosis"[MeSH] OR "tuberculosis"[tiab]** | 191904 |

**Table 2. Search methods for EMBASE conducted on 15 February 2013**

| **Number** | **Search Terms** | **Hits** |
| --- | --- | --- |
| #5 | **#3** AND **#4** | **25** |
| #4 | (**treatment** NEAR/3 (**adherence** OR **compliance**)):ab,ti OR (**therapy** NEAR/3 (**adherence** OR **compliance**)):ab,ti OR (**patient** NEAR/3 (**adherence** OR **compliance**)):ab,ti OR (**medication** NEAR/3 (**adherence** OR **compliance**)):ab,ti OR **'patient compliance'**/exp | **104990** |
| #3 | **#1** AND **#2** | **115** |
| #2 | **'mobile phone'**/exp OR (**mobile** NEXT/1 (**phone*** OR **telephone***)):ab,ti OR (**cellular** NEXT/1 (**phone*** OR **telephone***)):ab,ti OR (**cell** NEXT/1 (**phone*** OR **telephone***)):ab,ti OR **cellphone***:ab,ti OR **'reminder system'**/exp OR (**reminder** NEXT/1 **system***):ab,ti OR **'telemedicine'**/exp OR **telemedicine**:ab,ti OR **'wireless communication'**/exp OR (**wireless** NEXT/1 (**technology** OR **communication**)):ab,ti OR **'text messaging'**/exp OR (**text** NEXT/1 (**message*** OR **messaging**)):ab,ti OR **texting**:ab,ti OR (**messaging** NEXT/1 **service***):ab,ti OR **sms**:ab,ti OR **mms**:ab,ti OR **'medical informatics'**/exp OR **'mobile health'**:ab,ti OR **mhealth**:ab,ti | **45599** |
| #1 | **tuberculosis**:de OR **tuberculosis**:ab,ti | **217674** |

**Table 3. Search Terms for CENTRAL conducted on 15 February 2013**

| **Number** | **Search Terms** | **Hits** |
| --- | --- | --- |
| **#1** | "tuberculosis":ti,ab,kw | 2100 |
| **#2** | "cellular phone" or "reminder systems" or telemedicine or "wireless technology" or "text messaging" or text* or "medical informatics applications" or SMS or MMS or "mobile phone" or mHealth or "mobile health":ti,ab,kw | **3031** |
| **#3** | “medication adherence” or “patient compliance” or adherence or compliance:ti,ab,kw | **17705** |
| **#4** | #1 AND #2 AND #3 | **4** |

**Table 4. Search methods for CINAHL conducted on 15 February 2013**

| **Number** | **Search Terms** | **Hits** |
| --- | --- | --- |
| **#4** | #1 AND #2 AND #3 | 1 |
| **#3** | MH "Medication adherence" OR MH "Patient compliance" OR TI Adherence OR AB Adherence OR TI Compliance OR AB Compliance | 33593 |
| **#2** | MH "Cellular telephone" OR MH "Text messages" OR MH Wireless OR MH "Text messages" OR MH “Mobile phone” OR MH "Medical informatics" OR TI "Mobile phone" OR AB "Mobile phone" OR TI mHealth OR AB mHealth OR TI "mobile health" OR AB "mobile health" OR TI “Reminder Systems” OR AB “Reminder Systems” OR TI Telemedicine OR TI Telemedicine OR TI SMS OR AB SMS OR TI MMS OR AB MMS | 2903 |
| **#1** | MH Tuberculosis OR TI Tuberculosis OR AB Tuberculosis | 8533 |

**Table 5. Search methods for ISI Web of Science conducted on 15 February 2013**

| **Number** | **Search Terms** | **Hits** |
| --- | --- | --- |
| #4 | #1 AND #2 AND #3 | 14 |
| #3 | TS=(Medication adherence) OR TS=(Patient compliance) OR TI =(Adherence) OR TI=( Compliance) | 65196 |
| #2 | TS=(cellular phone) OR TS=(reminder systems) OR TS=(telemedicine) OR TS=(wireless technology) OR TS=(text messaging) OR TS=(text*) OR TS=( medical informatics applications) OR TI=(SMS) OR TI=(MMS) OR TI=(mobile phone) OR TI=(mHealth) OR TI=(mobile health) | 198823 |
| #1 | TS=(Tuberculosis) OR TI=(Tuberculosis) | 83208 |

**Table 6. Search method for Africa Wide Information conducted on 15 February 2013**

| **Number** | **Search Terms** | **Hits** |
| --- | --- | --- |
| #4 | #1 AND #2 AND #3 | 8 |
| #3 | SM "Medication adherence" OR SM "Patient compliance" OR TI Adherence OR AB Adherence OR TI Compliance OR AB Compliance | 10855 |
| #2 | SM "Cellular telephone" OR SM "Text messages" OR SM Wireless OR SM "Text messages" OR SM “Mobile phone” OR SM "Medical informatics" OR TI "Mobile phone" OR AB "Mobile phone" OR TI mHealth OR AB mHealth OR TI "mobile health" OR AB "mobile health" OR TI “Reminder Systems” OR AB “Reminder Systems” OR TI Telemedicine OR TI Telemedicine OR TI SMS OR AB SMS OR TI MMS OR AB MMS | 1128 |
| #1 | SM Tuberculosis OR TI Tuberculosis OR AB Tuberculosis | 136811 |

**Table 7. Search methods for WHOLIS conducted on 15 February 2013**

| **Number** | **Search Terms** | **Hits** |
| --- | --- | --- |
| #1 | mHealth | 1 |
